# Critical Illness Myopathy: Diagnostic Approach and Resulting Therapeutic Implications

**DOI:** 10.1007/s11940-022-00714-7

**Published:** 2022-03-28

**Authors:** Belén Rodriguez, Lars Larsson, Werner J. Z’Graggen

**Affiliations:** 1 Department of Neurosurgery, Inselspital, Bern University Hospital, University of Bern, 3010 Bern, Switzerland; 2 Section of Clinical Neurophysiology, Department of Clinical Neuroscience, Karolinska Institutet, 171 77 Stockholm, Sweden; 3 Department of Physiology and Pharmacology, Karolinska Institutet, 171 77 Stockholm, Sweden; 4 Viron Molecular Medicine Institute, Boston, MA 02108 USA; 5 Department of Neurology, Inselspital, Bern University Hospital, University of Bern, 3010 Bern, Switzerland

**Keywords:** Muscle velocity recovery cycles, Electromyography, Electrophysiology, Intensive care unit, ICU–acquired weakness, Myosin:actin ratio

## Abstract

**Purpose of review:**

Critical illness myopathy (CIM) is a common neuro-muscular complication of intensive care treatment associated with increased morbidity and mortality. The current guidelines for diagnosis include clinical and electrophysiological criteria as well as a muscle biopsy, and allow diagnosis only at an advanced stage of the disease. To date, there is no treatment for CIM available, apart from symptomatic and rehabilitative interventions. In this review, we discuss different diagnostic approaches and describe new treatment possibilities for CIM.

**Recent findings:**

Of the diagnostic approaches evaluated, a new electrophysiological technique for measuring muscle excitability has the greatest potential to allow earlier diagnosis of CIM than the current guidelines do and thereby may facilitate the conduction of future pathophysiological and therapeutic studies. Although clinical trials are still lacking, in animal models, BGP-15, vamorolone, and ruxolitinib have been shown to have anti-inflammatory effects, to reduce muscle wasting and to improve muscle function and survival.

**Summary:**

In recent years, promising methods for early and confirmatory diagnosis of CIM have been developed, but still need validation. Experimental studies on novel pharmacological interventions show promising results in terms of preventive CIM treatments, but future clinical studies will be needed to study the effectiveness and safety of these drugs.

## Introduction

During the last decades, survival rates of patients treated on intensive care units (ICUs) continuously improved. In parallel, complications of intensive care treatment have become more apparent and moved into the focus of research. Of these, neuro-muscular complications, especially critical illness myopathy (CIM), have received increasing interest because of their association with higher morbidity and mortality rates. Patients who develop CIM need to be treated longer on the ICU, require more intensive and longer rehabilitation, and have a reduced re-integration into former life. This impact has become even more evident during the COVID-19 pandemic, during which higher rates of CIM have been reported. This has resulted in an increasing number of patients with prolonged ICU stay, high occupancy of rehabilitation facilities, ultimately leading to significant challenges for the health care system when the required resources were scarce. This underlines the importance of further research into the pathophysiology underlying CIM and the development of preventive and therapeutic measures.

Before the term CIM was introduced and widely accepted, different names often including a description of clinical, pathophysiological, or histological characteristics were used, e.g., acute quadriplegic myopathy, thick filament myopathy, acute necrotizing myopathy of intensive care, acute corticosteroid myopathy, acute myopathy in severe asthma, and acute corticosteroid- and pancuronium-associated myopathy.

CIM is an acute primary myopathy that develops in critically ill patients. Clinically, the disease presents with flaccid paresis or plegia, which also involves respiratory muscles. Weakness is usually accompanied by pronounced atrophy of muscles. The histological hallmarks are a general decrease in muscle fiber cross-sectional area and a preferential loss of the motor protein myosin in the absence of inflammatory infiltrates but with detectable cytokine-activation [1–3]. First histological changes have been reported to occur already by day 5 of the ICU stay [4, 5] with a reduction of muscle fiber cross-sectional area by 4% per day during the early phase of the disease [6–8]. Another, but not uniformly reported, histological feature of CIM is muscle fiber necrosis [1, 6].

The exact incidence of CIM is unclear, which is mainly due to the different implemented diagnostic methods and the diagnostic criteria referred to [9]. Accordingly, with 9–86% a wide range of incidences has been reported [10–13]. A systematic review reported an approximate incidence of 40% [14]. The authors also showed that the incidence of failure of diagnostic assessment was higher using a purely clinical approach (26%) compared to an electrophysiological technique (2%). Recent studies in critically ill COVID-19 patients reported CIM incidences of 50–64% [15–17], thus indicating that the incidence of CIM may be higher in a COVID-19 population than in a non-COVID-19 population. CIM sometimes co-exists with critical illness polyneuropathy, which shows partially overlapping clinical symptoms and is associated with a worse prognosis [18]. In patients with sepsis, multi-organ failure, or protracted mechanical ventilation, the combined prevalence was estimated to be almost 50% [9, 19, 20]. However, recent literature suggests that the prevalence of critical illness polyneuropathy is far lower than previously thought [21].

The clinical outcome of critically ill patients who developed CIM is heterogeneous, but in general CIM correlates with long-term consequences for the patients and their families [22–24]. The development of CIM is associated with a 15–25% increase in mortality, both regarding in-hospital and 5-year mortality [23, 25–27]. The in-hospital mortality rate increases with the severity of muscle weakness, even after adjusting for severity of illness [28]. Van Aerde et al. recently reported that clinical as well as electrophysiological measures indicating muscle dysfunction were independently associated with increased 5-year mortality [29•]. Due to the arising treatment complications when a patient develops CIM and the resulting extension of ICU and hospital stay, health care costs increase by 30.5% [23]. One year after hospital discharge, 14% of critical illness survivors continue to show signs of muscle weakness; even after 2 years, 9% of survivors still have muscle weakness [30]. In a subgroup of patients who had acute respiratory distress syndrome, 50% of survivors experienced muscle weakness over a 5-year follow-up period, which was associated with lower survival [27]. Similarly, it has been described that 6 months after critical illness, patients who received mechanical ventilation and developed profound muscle weakness had lower health-related quality of life than patients without weakness, and that muscle strength positively correlated with physical functioning [31].

Although many studies investigated risk factors for developing CIM, the etiology still remains elusive. However, the development of CIM is assumed to be multifactorial. The risk factors that have previously been proposed and mostly looked at are the patients’ premorbid health-status, duration of intensive care treatment and mechanical ventilation, treatment with neuromuscular blocking agents or sedative medication, and the degree of severity of the acute disease, especially when multi-organ failure is involved [1, 32–34]. The latter is the most consistently shown risk factor in many studies [33, 35–37], which generated the assumption that CIM may be another manifestation of multi-organ dysfunction [33, 38]. Data on the possible negative impact of treatment with corticosteroids, neuromuscular blockers, or sedating drugs are inconsistent and sometimes even contradictory [9, 10, 37, 39–44]. Critically ill patients with severe sepsis and septic shock often have increased serum glucose levels. Van den Berghe et al. reported that intensive insulin therapy was associated with a lower incidence of CIM [45], but later it was shown that critically ill patients receiving intensive insulin therapy had a higher risk of severe hypoglycaemia and that the 90-day mortality rate was higher compared to those who had a liberal glucose control [46, 47]. Nevertheless, lower serum glucose and higher insulin levels seem to be protective factors for CIM [48–50]. More recently, results both from studies done in critically ill patients as well as from animal models highlighted that a profound systemic inflammatory response and factors related to bioenergetic failure such as microvascular, metabolic, and electrical muscle membrane alterations underlie the development of CIM [51, 52]. Muscle atrophy in CIM is a consequence partly of decreased protein synthesis and increased protein degradation, and is largely influenced by the ubiquitin–proteasome system (please see [52] for a more detailed description).

## Clinical presentation and diagnosis

CIM becomes clinically apparent in the subacute phase of critical illness. Once the patient stabilizes from acute illness and analgosedation can be reduced, muscle weakness and/or weaning difficulties are noticed. The neurological examination typically reveals muscle atrophy and symmetrical flaccid paresis. Craniofacial muscles are typically spared or less affected. Furthermore, deep tendon reflexes are reduced or rarely abolished. Detailed testing of the sensory systems is often difficult in ICU patients, but will be within normal limits.

The current guidelines for diagnosis of *definite* CIM include clinical and electrophysiological criteria as well as a muscle biopsy [1]. The two clinical criteria require (i) a positive history for critical illness (multi-organ dysfunction and failures) and (ii) limb weakness or difficulty in weaning the patient from the ventilator, not caused by non-neuromuscular etiologies. The electrophysiological criteria depend on motor and sensory nerve conduction studies of at least two nerves and additional needle electromyographies. In motor nerve conduction studies, amplitudes of compound muscle action potentials are reduced by more than 20% of the lower limit. The duration of compound muscle action potentials can be prolonged, but has also been reported to be normal [53, 54•]. Additionally, nerve conduction blocks must be excluded. Sensory nerve conduction studies are within normal limits or only slightly reduced in amplitude (less than 20% of lower limits). Needle electromyography typically reveals motor unit potentials of short duration and low amplitude if the patient is able to cooperate. Spontaneous activity (fibrillation potentials and positive sharp waves) can be present. If recruitment can be tested, it will be normal. In unconscious or non-cooperative patients, direct muscle stimulation can be performed as an additional test. The comparison of the elicited compound muscle action potential due to nerve and direct muscle stimulation via a monopolar needle electrode shows reduced or absent responses of both stimulation modalities [55, 56]. Finally, a neuromuscular transmission deficit has to be excluded with repetitive motor nerve stimulation. The results of muscle biopsy have already been reported above. Instead of an open muscle biopsy, less invasive fine needle biopsy can be performed to measure the myosin:actin ratio [57•]. Ratios < 1.7 have been reported as abnormal. As already mentioned, diagnosis of *definite* CIM requires that all criteria are fulfilled. If patient examination is not possible and/or muscle biopsy cannot be performed, but all other criteria are fulfilled, diagnosis of *probable* CIM is made. If only the clinical criteria are considered, the diagnosis of *ICU*–*acquired weakness* is used.

In recent years, a growing number of studies have abandoned the use of this elaborated multimodal approach for diagnosis of *definite* CIM and have instead focused on the endpoint *ICU*–*acquired weakness*. Consequently, more specific recommendations for diagnosis of *ICU*–*acquired weakness* have emerged, which demand the assessment of muscle strength using the Medical Research Council (MRC) score for 12 muscle groups: shoulder abduction, elbow flexion, wrist extension, hip flexion, knee extension, and ankle dorsiflexion of both sides. A sum score < 48 is required for diagnosis of *ICU*–*acquired weakness* [58, 59]. Nevertheless, *ICU*–*acquired weakness* is a purely clinical diagnosis and therefore omits determining the exact etiology of weakness allowing for a broad differential diagnosis, including critical illness polyneuropathy, Guillain-Barré syndrome, Myasthenia gravis, and myositis in addition to CIM [60]. This indicates that the diagnosis of *ICU*–*acquired weakness* is not suitable as an outcome measure for the conduction of interventional and pharmacological trials, since CIM and the other etiologies differ in pathophysiology, prognosis, recovery, and potential treatment.

Besides the complex and invasive approach, the current diagnostic criteria for CIM also bear the disadvantage of allowing diagnosis only at an advanced stage when muscle damage has already predominantly occurred. Hence, they cannot be used for screening and identifying patients at risk or to monitor disease progression from the early phase onwards and are thus not suitable for conducting preventive and therapeutic trials.

In 1971, Cunningham et al. invasively measured absolute muscle membrane potential in 21 severely ill patients and showed for the first time that their muscle membranes were depolarized (resting membrane potential − 66.3 ± 9.0 mV, versus − 88.8 ± 3.8 mV in 26 healthy volunteers (mean ± SD)), which was paralleled by an increase in intracellular Na^+^ concentration [61]. Although CIM was not a known disease at the time, this study was the basis for the later hypothesis that changes in electrical properties precede structural changes and are the first sign of the evolving disease and therefore a tool for early diagnosis and disease monitoring. Only in 2008, Allen et al. performed single muscle fiber recordings in patients with CIM and found a marked slowing of muscle-fiber conduction velocities, increased refractoriness as a possible sign of muscle membrane depolarization, and occurrence of muscle-fiber conduction block [53]. The latter is probably the reason for the sometimes described finding of muscle inexcitability due to direct muscle stimulation [62, 63]. In a rat model of CIM, altered excitability of muscle membrane was attributed to a hyperpolarized shift in the voltage dependence of inactivation of Nav1.4 sodium channels [64–66]. This explains why muscle membrane depolarization in CIM is related to an important loss of available sodium channels, thereby reducing muscle fiber excitability. In an in vitro study, Haeseler et al. [67] identified that a lipopolysaccharide endotoxin from *Escherichia coli* interacts with Nav1.4 Na^+^ channel alpha-subunits and reduces Na^+^ channel availability only at depolarized resting potentials [67].

Since single muscle fiber recordings are generally very difficult to conduct and time consuming, and therefore not an ideal diagnostic tool, a new technique for measuring muscle excitability has been developed [68, 69]. This technique uses direct needle muscle stimulation to excite a small cluster of muscle fibers, from which recordings are made with a concentric EMG electrode [70]. After a first evoked action potential, the excitability of the membrane for a second action potential depends on the interstimulus interval. If the interstimulus interval is very short, the membrane will be in- or hypoexcitable (= absolute or relative refractory period). During the relative refractory period, an action potential can only be elicited with higher stimulation intensities and will propagate with a slower conduction velocity. The phase of refractoriness is followed by a second phase of altered excitability, which is determined by the principle that an elicited muscle action potential is followed by a depolarizing afterpotential. This afterpotential depends on the charge left on the capacitance of the membrane [71]. Hence, a second stimulus applied during the period of the afterpotential will propagate faster along the muscle membrane (= phase of supernormality). The afterpotential itself and also the refractory period strongly depend on the membrane potential. Multi-fiber muscle velocity recovery cycle measurements allow to assess alterations of refractoriness and supernormality, and thus can be used to detect relative changes of muscle membrane potential [68, 69]. In patients with the diagnosis of *probable* CIM, this technique confirmed changes related to either muscle fiber membrane depolarization and/or, as elaborated above, to heightened sodium channel inactivation [54•, 72]. Two studies using a porcine model of sepsis found similar alterations within 6 h of sepsis onset, indicating that muscle membrane changes may indeed represent an early sign of evolving CIM [73], and a dependence of muscle excitability alterations from sepsis induced changes of microcirculation [74]. A recently conducted prospective cohort study in patients with COVID-19–associated acute respiratory distress syndrome found that muscle excitability measurements recorded 10 days after intubation discriminated between patients who, according to the diagnostic criteria, developed CIM and those who did not, with a diagnostic accuracy of 90%. Furthermore, this study confirmed that muscle membrane depolarization develops very early in the course of CIM. Muscle excitability parameters measured within 24 and 48 h after intubation discriminated between patients who will develop CIM and those who will not with 73% and 82% diagnostic accuracy, respectively. The findings of this study provide further evidence that muscle excitability measurements are a promising technique for early and confirmatory diagnosis of CIM. Regarding the proposed risk factors, the study could not confirm that treatment with neuromuscular blocking agents or any other medication (sedative and vasoactive drugs, glucocorticoids) is associated with the development of CIM, but found that patients who developed CIM were mechanically ventilated longer and had generally a higher disease severity than patients who did not. Interestingly, patients who developed CIM had higher serum potassium levels and variability throughout the first 10 days of ICU stay and a higher incidence of renal failure [Rodriguez et al. under review].

## Treatment options

To date, there is no available treatment for CIM, apart from symptomatic and rehabilitative interventions. This highlights the importance of developing and implementing preventive strategies during intensive care and early recognition of patients who are at increased risk for developing CIM. The treatment of the disease underlying critical illness, e.g., treatment of severe infection and multi-organ failure, is the central component in preventive treatment for CIM. A Cochrane review of interventional trials reported that intensive insulin therapy moderately reduced the prevalence of CIM and critical illness polyneuropathy in two studies (see above) [11, 45, 75]. Recently, a new preventive treatment possibility has been described and investigated: Cacciani et al. [76•] reported that treatment with the chaperone co-inducer BGP-15, a heat shock protein and insulin-sensitizer drug candidate, may protect muscle fiber force and improve survival within the first 5 days of critical illness. At longer durations, e.g., 8 to 10 days, BGP-15 no longer had a protective effect when preferential loss of myosin, the hallmark of CIM, had become manifest [76•]. Vamorolone represents a new class of dissociative glucocorticoids with the same anti-inflammatory effects as prednisolone, but fewer harsh negative hormonal effects on muscle tissue and originally designed to treat patients with Duchenne muscular dystrophy [77]. In experimental studies, vamorolone reduced the loss of muscle mass, myosin loss, and muscle function in response to 5 days mechanical ventilation and immobilization [78•]. The JAK/STAT inhibitor ruxolitinib attenuated the negative effects of the ICU condition similar to BGP-15 and vamorolone [79•]*.* BGP-15, vamorolone, and ruxolitinib all have anti-inflammatory effects, albeit at different levels of inflammatory pathways, and in addition to improving muscle function, reducing muscle wasting, and myosin loss, they all improved survival in an experimental ICU model where rats were exposed to immobilization and neuromuscular blockade for 5 days and longer [76•, 78•].

## Conclusion and future directions

Constant improvements in modern intensive care have led to higher survival rates of patients, but also brought complications of intensive care treatment, such as CIM, into focus. Patients who develop CIM need longer intensive care, stay longer in hospital, and have a higher need for rehabilitation. CIM is not only associated with increased morbidity and mortality rates, but also reduced re-integration in former life. Consequently, not only the health-care cost for patients with CIM is significantly higher, but patients and their family members also have to endure high long-term socioeconomic burdens. The increase in CIM incidence during the COVID-19 pandemic has made this impact even more evident, and thus underlines the importance of further research into the pathophysiology underlying CIM, and the development of preventive and therapeutic measures. In recent years, promising methods for early diagnosis of CIM have been developed, but still need validation in larger patient cohorts. Tools for early diagnosis are of very high importance and form the basis for future studies, which aim to further investigate the pathophysiology of the disease and for the monitoring of therapeutic studies. Novel pharmacological interventions have been evaluated in experimental models (see above) showing promising preventive effects, but future clinical studies will be needed to study the effectiveness and safety of these drugs.
